# Prefrontal Cerebral Oxygenated Hemoglobin Concentration during the Category Fluency and Finger-Tapping Tasks in Adults with and without Mild Cognitive Impairment: A Near-Infrared Spectroscopy Study

**DOI:** 10.3390/brainsci12121636

**Published:** 2022-11-29

**Authors:** Shingo Takahashi, Yosuke Tomita, Shigeya Tanaka, Noriko Sakurai, Naoki Kodama

**Affiliations:** 1 Department of Healthcare Informatics, Faculty of Health and Welfare, Takasaki University of Health and Welfare, 37-1 Nakaorui-machi, Takasaki 370-0033, Japan; 2 Department of Physical Therapy, Faculty of Health Care, Takasaki University of Health and Welfare, Takasaki 370-0033, Japan; 3 Department of Radiological Technology, Faculty of Medical Technology, Niigata University of Health and Welfare, 1398 Shimami-cho, Kita-ku, Niigata 950-3198, Japan

**Keywords:** near-infrared spectroscopy, mild cognitive impairment, finger tapping, category fluency task, dual task

## Abstract

Mild cognitive impairment (MCI) is considered to be the limit between the cognitive changes of aging and early dementia; thus, discriminating between participants with and without MCI is important. In the present study, we aimed to examine the differences in the cerebral oxyhemoglobin signal between individuals with and without MCI. The cerebral oxyhemoglobin signal was measured when the participants (young and elderly controls as well as patients with MCI) performed category fluency, finger tapping, and dual tasks using head-mounted near-infrared spectroscopy; the results were compared between the groups. The cerebral oxyhemoglobin signal trended toward the highest values during the category fluency task in young participants and during the finger-tapping task in elderly participants regardless of the MCI status. The area under the curve was approximately 0.5, indicating a low discrimination ability between elderly participants with and without MCI. The measurement of the blood flow in the prefrontal cortex may not accurately quantify cognitive and motor performance to detect MCI. Finger tapping may increase cerebral blood flow in individuals with and without MCI during the task.

## 1. Introduction

People with dementia (major neurocognitive disorders) suffer from cognitive decline and daily life disabilities. Dementia is characterized by (i) significant cognitive impairment, such as memory disorder [1], (ii) daily living dependence [2], and (iii) distinguishable symptoms from other psychological disorders or delirium [3]. Most of the etiologies of dementia (e.g., Alzheimer’s disease) are progressive, which increases the care needs of the affected individuals [4]. Dementia symptoms include poor recall, losing items, wandering, hoarding, repetitive mannerisms and activities, hallucinations, and delusions. Experiencing changes in personality and behavioral difficulties may develop as the disease progresses [5,6]. The detection of early-stage dementia and the initiation of early cognitive training are important for delaying symptom progression. Mild cognitive impairment (MCI) is considered to be the borderline between the cognitive changes of aging and early dementia [7], and the discrimination between MCI and non-MCI is important.

In a previous study, the cerebral oxyhemoglobin signals were measured in 21 healthy volunteer students and 50 patients with dementia using near-infrared (NIR) spectroscopy [8]. The advantages of NIR spectroscopy include relative insensitivity to movement, portability, and affordability, which permit the designing of tasks for assessing populations, such as children or older people. They found that during the category fluency task, healthy young participants presented with increased blood flow; in contrast, participants with dementia demonstrated no changes in the prefrontal blood flow [8]. In another study, the authors reported that during the category fluency tasks, healthy elderly controls demonstrated a higher cerebral oxyhemoglobin signal in the left cerebral hemisphere compared to patients with dementia [9]. A previous study reported that compared to young controls, patients with dementia had decreased cerebral blood flow during cognitive tasks [8].

Prior studies have shown that performing cognitive and motor tasks, such as verbal fluency and finger-tapping tasks, is useful in detecting cognitive decline. Cognitive and executive functions as well as verbal motor functions are related to the performance of the category fluency task [10,11]. Considering the sub-second repetitive motor timing, such as in the finger-tapping task, executive control and working memory were involved [12,13]. Furthermore, recent studies have reported a correlation between the finger-tapping task and cognitive ability in MCI detection [14,15]; another study reported the usefulness of dual tasks in MCI screening [16]. Thus, a dual task combining category fluency and a finger-tapping task could be a sensitive task for characterizing altered brain activity in individuals with MCI.

However, the usefulness of the oxyhemoglobin signal during verbal fluency and finger-tapping tasks, as well as their combination to characterize MCI, remains unclear. The measurement of the oxyhemoglobin signal during these tasks may discriminate between MCI and healthy elderly participants. This study aimed to examine the extent to which age and MCI affect change in oxyhemoglobin using NIR spectroscopy during category fluency, finger-tapping, and dual tasks. Additionally, the effectiveness of cerebral oxyhemoglobin signal measurements in distinguishing between elderly participants with and without MCI was examined. 

## 2. Materials and Methods

### 2.1. Participants and Methods

This study was a cross-sectional study that involved only community-dwelling older adults and included 102 elderly patients and 30 young controls. The Japanese version of the Montreal Cognitive Assessment (MoCA-J) was used to discriminate between elderly participants with (MCI group) and without MCI (healthy elderly group), with a cutoff value of 26 points. A value of ≥26 points indicated that the individual was healthy, while a value of <26 points was defined as having MCI [17]. Among the 102 elderly patients, 41 were included in the group of elderly participants without MCI (nine men and 32 women), and 61 were included in the MCI group (19 men and 42 women) (Table 1). Among the 30 young controls, 14 were men and 16 were women. Those with a diagnosis of dementia or neurodegenerative diseases, or those who were those comorbidities were excluded. The sample size was calculated using the mean difference of the oxyhemoglobin signals between cognitive tasks reported in a previous study. The result of the sample size calculation showed that 15 participants were required in each group [9]. The study was approved by the Research Ethics Committee of Takasaki University of Health and Welfare and was conducted in accordance with the Declaration of Helsinki. Written informed consent was obtained from all participants prior to their participation in the study.

**Figure 1:**
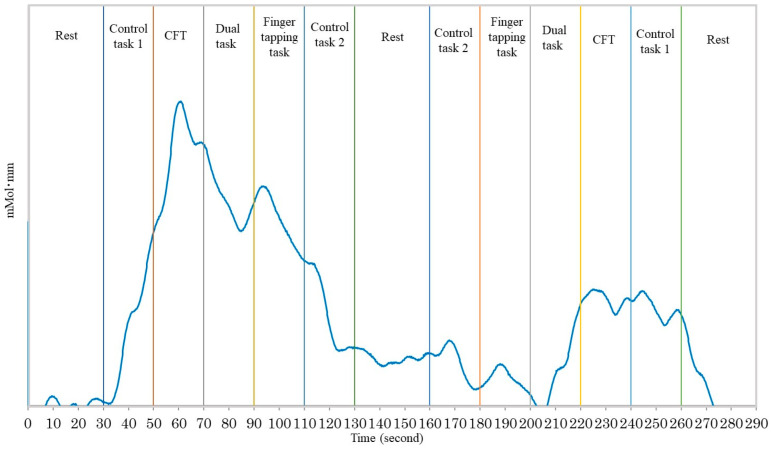
The measurement tasks included the category fluency task, finger-tapping task, dual task with both tasks, control task 1, and control task 2. Control task 1 involved pronunciation of the Japanese characters “a, i, u, e, o”. Control task 2 involved keeping the index finger and thumb together.

An NIR spectroscopy system (WOT100, Hitachi High-Technologies Corporation, Tokyo, Japan) was used to examine the prefrontal oxyhemoglobin signal of the brain while the participants performed the three types of tasks. The NIR spectroscopy system comprised a computer, control box, and headset. The oxyhemoglobin, deoxyhemoglobin, and total hemoglobin levels were measured using this device at 200 Hz. The measurement protocol is shown in Figure 1. NIRS measurements are often averaged by setting up a block design, and in this study, each task was set up twice. Each task lasted 20 s, with the first 5 s announcing to the participants to perform the task and the following 15 s spent on performing the task. The measurements of deoxy- and oxyhemoglobin were performed using wavelengths of 705 nm and 830 nm, respectively. The oxyhemoglobin levels were primarily analyzed for each task in our study. In addition, NIR spectroscopy data were used for receiver operating characteristic analysis to analyze the ability of oxyhemoglobin to discriminate between elderly participants with and without MCI [18].

### 2.2. Category Fluency Task

The verbal fluency task is a well-established neuropsychological test of the frontal lobe; previous functional magnetic resonance imaging and NIR spectroscopy studies have demonstrated this task’s ability to stimulate the prefrontal cortex [19]. The verbal fluency task includes two types of tasks: the category and letter fluency tasks. The category fluency task involves naming the largest possible number of members of a category within a limited time period. The category fluency tasks are used to evaluate executive and language functions and semantic memory [20]. A verbal fluency task involves naming the largest possible number of words in a specific category within a limited time period. In this experiment, we used a category fluency task. The categories used were “vegetables” and “fruits.” Further, the participants pronounced the Japanese characters of “a, i, u, e, o” before and after the task (control task 1). The measurement protocol was based on previous studies [21,22,23]. The performance of the category fluency task was quantified using the average of the two responses.

### 2.3. Finger-Tapping Task

**Figure 2:**
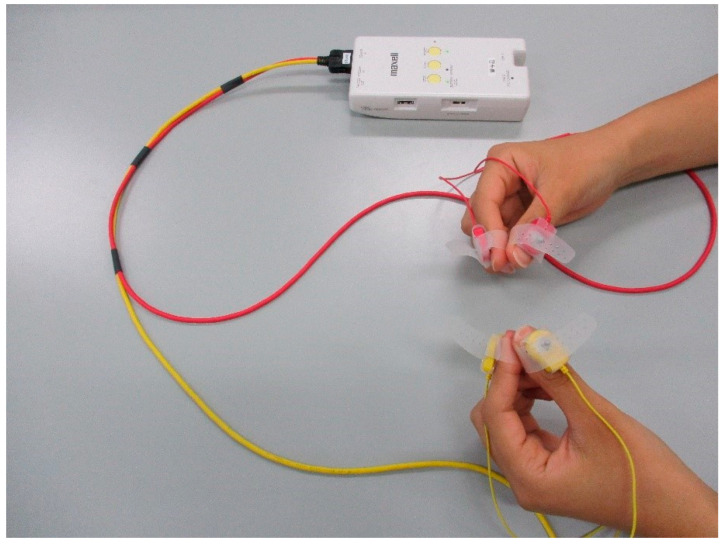
Control task 2 condition. Control task 2 involved keeping the index finger and thumb together.

Bimanual finger-tapping was performed for 15 s as quickly and widely as possible [24]. The distance between the thumb and index finger during the finger-tapping movement was measured using a magnetic sensor at 100 Hz (UB-2, Maxell, Ltd., Tokyo, Japan). The tapping of the participants was alternated between both hands, where the fingers on one hand were closed while those on the other side were opened (when the thumb and index finger of the right hand were open, the thumb and index finger of the left hand were in an attached position). The finger-tapping task was performed twice, and the average tap count was obtained. Further, the participants kept the index finger and thumb attached to each other on both hands before and after the finger-tapping task (control task 2). Control task 2 and the mounted UB2 are shown in Figure 2.

### 2.4. Dual Task

The dual task is the simultaneous performance of two tasks, such as cognitive and motor tasks. Different combinations of motor tasks and cognitive exercises should be explored [16]. A previous study indicated that dual-task testing may be useful in differentiating between dementia and other cognitive impairment diagnoses [25]. In this experiment, finger-tapping and verbal fluency tasks were employed as dual tasks. 

### 2.5. Near-Infrared Spectroscopy

**Figure 3:**
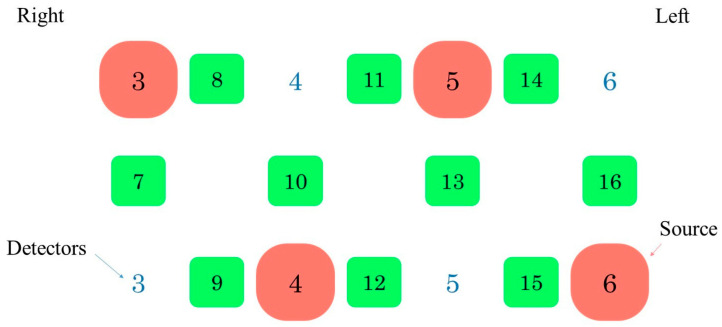
Probe set of WOT100. WOT100 has 10 measurement channels in the prefrontal cortex. Channels 7, 8, 9, and 10 are in the right prefrontal cortex region while channels 13, 14, 15, and 16 are in the corresponding left region. The light-emitting diode and light-receiving element are shown by 3–6.

The NIR spectroscopy principle is based on the modified Lambert–Beer law. NIR spectroscopy monitors NIR light absorption by oxyhemoglobin and deoxyhemoglobin using two different wavelengths [26]. The modified Lambert–Beer law is expressed as follows:

$$-\log\left(\frac{I}{I_{0}}\right)=\left(c\times \varepsilon_{\lambda }\times l\times DPF\right)+G$$

where *DPF* is the differential path length factor accounting for the nonlinear light trajectory in biological media, and *G* is the scatter [27]. *I_0_* is the incident light intensity, *I* is the transmitted light intensity, *ε* is the molar absorption coefficient, *C* is the concentration of the substance absorbing the light, and *l* is the distance between the point of incident and transmitted light. Brain activity is associated with several physiological processes, particularly neurovascular coupling, and is known to be assessed in the optical window of 650–950 nm NIR light [28]. WOT100 measurements were performed using a 10-channel system in the prefrontal cortex (Figure 3). Channels 7–10 and 13–16 were designated as the right and left channels, respectively. This device is able to detect changes in the oxyhemoglobin, deoxyhemoglobin, and total hemoglobin levels in the prefrontal area. The optodes of WOT100 are arranged in an alternating geometry with an inter-optode separation of 3 cm, creating 16 source-detector combinations. To shield the headset from the surrounding light, a shading cap was used [29].

### 2.6. Data Analysis and Statistics

Linear trend components were eliminated from the obtained waveform. NIR spectroscopy data are expressed as means ± standard deviations. In this case, motion artifacts were not removed as the tasks employed cognitive and finger-tapping tasks, and there were no significant body movements. To compare the NIR spectroscopy data between participants, the measured signals were normalized by Z-score transformation [30,31]. We verified the normal distribution of the data using the Shapiro–Wilk test. The mean and standard deviation during the 1 s before the start of each task were used to normalize the signal. To evaluate brain activation, the average of the normalized oxyhemoglobin signal values in each task was calculated. Further, the task was arranged such that each task condition was repeated twice by each participant, where the order of conditions was changed for each participant. Finally, the average data of the two trials were used for the statistical analysis. Moreover, values greater than the mean ±2 standard deviations were eliminated as outliers.

We tested for the differences in the oxyhemoglobin levels between the tasks (rest, CFT, dual task, and FT) using a one-way analysis of variance and Bonferroni multiple comparison test, where statistical significance was corrected by dividing the significance level with the total number of tasks (i.e., corrected alpha level being 0.05/4 = 0.0125). A *p*-value < 0.0125 was considered statistically significant. The area under the curve (AUC) of the receiver operating characteristic (ROC) curve was calculated to estimate the validity of the oxyhemoglobin signal during the category fluency task, dual task, and finger-tapping task to distinguish elderly participants with and without MCI. We used SPSS software (version 27.0 for Windows; IBM Corp., Armonk, NY, USA) for the statistical analyses and calculation of AUC.

## 3. Results

**Figure 4:**
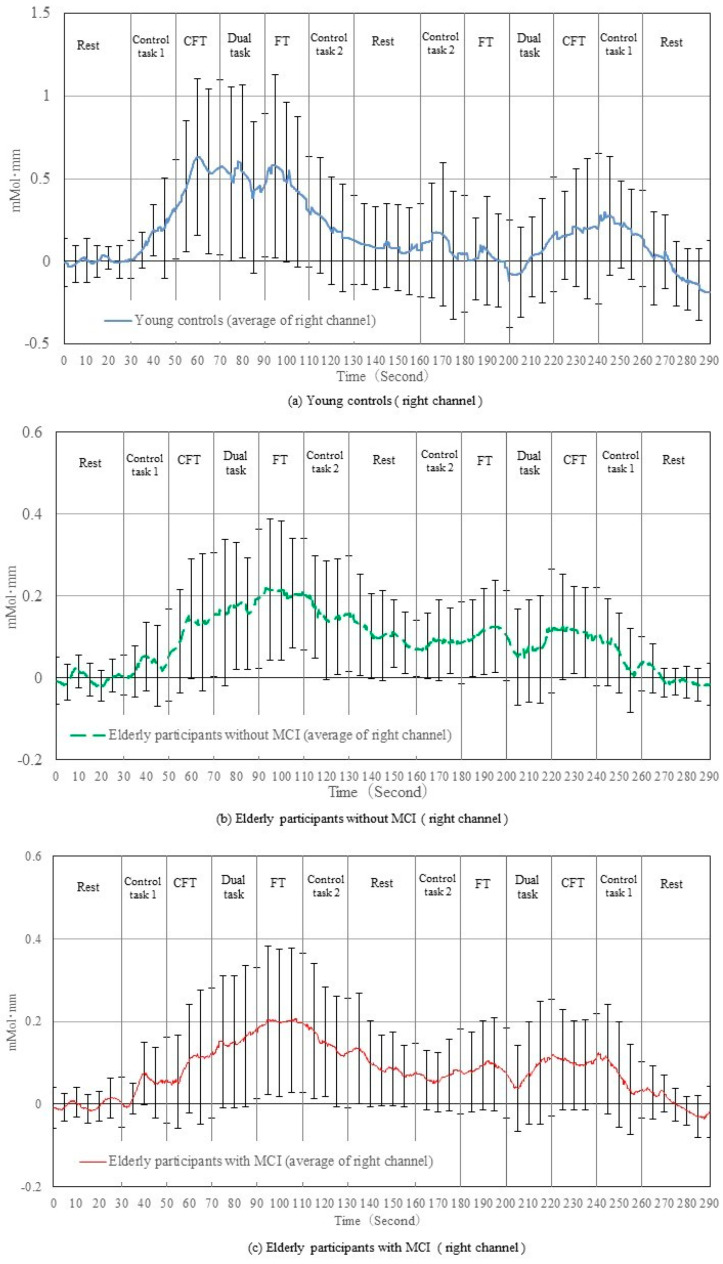
Changes in raw oxyhemoglobin levels of the right channels before normalization. Representative data from young controls (**a**), elderly participants without mild cognitive impairment (**b**), and elderly participants with mild cognitive impairment (**c**) are shown.

**Figure 5:**
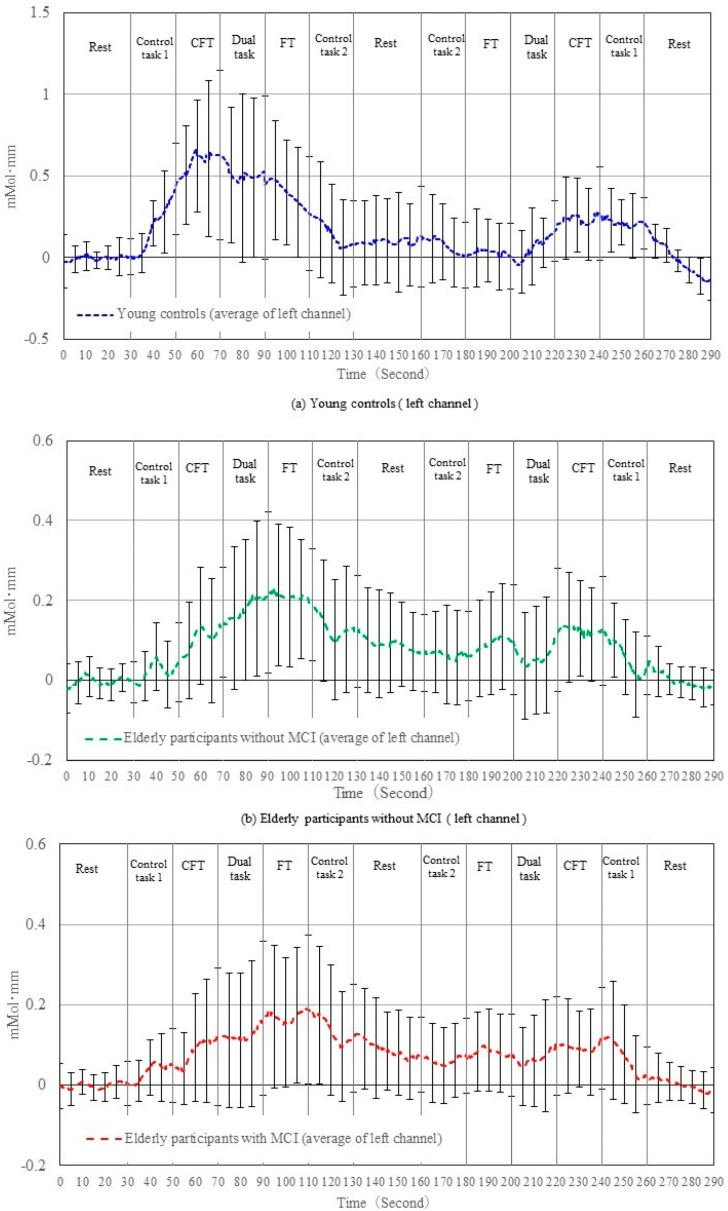
Changes in raw oxyhemoglobin levels of the left channels before normalization. Representative data from young controls (**a**), elderly participants without mild cognitive impairment (**b**), and elderly participants with mild cognitive impairment (**c**) are shown.

Figure 4 and Figure 5 show the changes in the raw oxyhemoglobin signals in the right and left prefrontal cortices, respectively, during the experiment in elderly participants with and without MCI and young controls. In the category fluency task on the first trial, the oxyhemoglobin signals in all three groups showed an increasing trend at the beginning of the task, and then the signal was maintained or decreased toward the end of the task on the second trial. In the dual task, the oxyhemoglobin signals in young controls showed a decreasing trend after the start of the task, followed by a slight increase in the first trial; whereas in the second trial, the signals substantially increased at the beginning of the second trial and slightly decreased after the start of the task, followed by a slight increase. However, the oxyhemoglobin signals of the elderly participants with and without MCI were maintained or showed a small increasing trend from the previous task on both the first and second trials. In the finger-tapping task, the oxyhemoglobin signals in young controls showed a trend toward a decrease in both the first and second trials, while it was maintained or increased in the elderly participants with and without MCI. The signals obtained from the right channels were similar to those from the left channels. 

**Figure 6:**
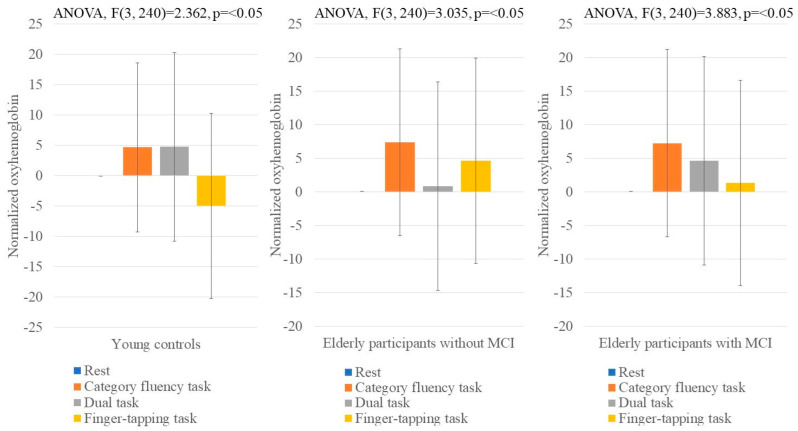
Condition-specific differences in normalized oxyhemoglobin signal of right channels. Figure 6 shows the normalized oxyhemoglobin signal for each task in the right channel. The blue color represents data from rest (average of right channel). The deep orange color represents data from the category fluency task (average of right channel). The gray color represents data from dual tasks (category fluency task and finger-tapping task; average of right channel). The light orange color represents data from the finger-tapping task (average of right channel).

**Figure 7:**
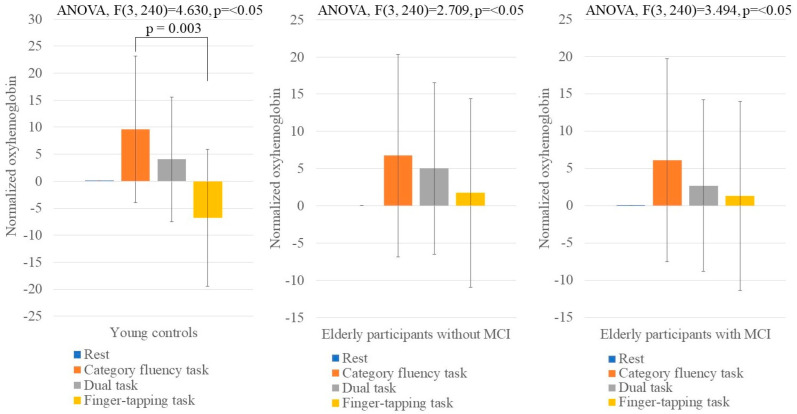
Condition-specific differences in normalized oxyhemoglobin signal of left channels. Figure 7 shows the normalized oxyhemoglobin signal for each task in the left channel. The blue, deep orange, gray, and light orange colors represent data from rest, the category fluency task, dual task, and finger-tapping task, respectively.

After verifying the trend of changes in the raw oxyhemoglobin signals, the normalized oxyhemoglobin signals were used to compare the group differences in the prefrontal brain activity during each task. The result of the one-way analysis of variance and Bonferroni multiple comparison test by normalized oxyhemoglobin levels during each task are shown in Figure 6 (on the right channels) and Figure 7 (on the left channels). In all the groups, the repeated analysis of variance results show significant differences between the tasks (Figure 6 and Figure 7), while the post-hoc analysis results show that only young controls showed significant differences between the category fluency and finger-tapping tasks (Figure 7; *p* = 0.003). When comparing the hemoglobin levels in the elderly with and without MCI, the AUC ranged from 0.411 to 0.570 (Table 2).

The area under the curve (elderly participants with MCI/without MCI) ranged from 0.411 to 0.570.

## 4. Discussion

The brain hemoglobin levels of the elderly and young patients were measured while they performed category fluency, finger-tapping, and dual tasks. The changes in the oxyhemoglobin signal were similar between participants with and without MCI; however, they were different from those in the young participants. The right channel showed results similar to the left channel. The AUC value was approximately 0.5, and the accuracy of discrimination between the elderly with and without MCI was low. 

Age may affect the type of task that induces changes in the prefrontal oxyhemoglobin signal. Specifically, young participants had a higher oxyhemoglobin signal during category fluency than the elderly participants did, as shown in Figure 4 and Figure 5. This result is consistent with those of previous studies that have demonstrated decreased oxyhemoglobin signal changes in elderly individuals compared to young individuals, which were further decreased in patients with dementia [8,32]. In contrast, young individuals showed a decreased oxyhemoglobin signal during the finger-tapping task. Previous studies have shown that the frontal lobe, the designated measurement site of our study, has been significantly stimulated by category fluency tasks since it involves memory, language function, and executive function [33,34]. Our study results suggest that the prefrontal hemodynamics during the finger-tapping task were significantly lower than during the category fluency task in young adults (Figure 7), likely because the performance of the task was simple for young adults. In contrast, the oxyhemoglobin signal during the finger-tapping task showed little difference compared to the other tasks in the elderly. This may be attributed to the higher rate of change in the regional cerebral hemoglobin during maximal-effort finger tapping compared to the low-effort finger-tapping tasks, suggesting that the finger-tapping task was cognitively demanding for the elderly [35].

Contrary to our prediction, changes in the oxyhemoglobin signal during the dual task were not significantly different from the other tasks in both the young and elderly with and without MCI. The dual task comprised both category fluency and finger-tapping tasks, which activated a wide range of cortical areas, including the prefrontal cortex and primary motor cortex [19,36]. A previous study found an increase in the prefrontal cortex during the dual task of cognitive tasks and walking [37]. However, Watanabe reported a negative relationship between prefrontal memory-related neural activity and attention difficulty during dual tasks [38]. It has been shown that when resources for information processing are limited, activities limit each other as they compete for resources [39]. The dual task of the finger-tapping task and the category fluency task may have been difficult and, therefore, such competition for cognitive resources may have occurred in elderly participants. This may explain the greater changes in the oxyhemoglobin signal in healthy young people during the category fluency task (Figure 4), and decreased performance of the task in elderly participants with and without MCI during the finger-tapping task of the dual task (Table 1).

Furthermore, the comparisons between the oxyhemoglobin levels of the elderly participants with and without MCI show that the AUC ranged from 0.411 to 0.570, suggesting the inability of the hemoglobin level during the tasks to discriminate between older individuals with and without MCI. Previous studies have reported differences in the changes in the oxyhemoglobin signal between patients with dementia and healthy controls [40,41,42]. A previous study found evident differences in the performance of both cognitive (i.e., verbal fluency task) and motor (i.e., finger-tapping task) performance between individuals with and without MCI [15]. These results suggest that patients with dementia may be characterized by a large decrease in brain activity. However, the present study demonstrated that the oxyhemoglobin signal remained similar between elderly participants with and without MCI. The results of this study suggest that although finger tapping may have increased cerebral blood flow in the participants with and without MCI during the task, the measurement of the oxyhemoglobin signal may not have provided additional benefits in quantifying the cognitive and motor performance in detecting MCI. Although the causal relationship between reduced cerebral blood flow and the development of dementia is not clear, a previous study demonstrated that reduced cerebral blood flow is closely related to the onset and exacerbation of cognitive status [43]. There may be a substantial difference in cerebral hemodynamics between elderly individuals with MCI and those with advanced dementia.

This study has certain limitations. First, only the oxyhemoglobin level in the prefrontal cortex was examined. As motor tasks were performed, the parietal lobe should have been examined as well. Although we focused on changes in oxidized hemoglobin, changes in deoxygenated hemoglobin and total hemoglobin may provide additional information in the characterization of MCI. Future studies should investigate the relationship between changes in oxidized hemoglobin and task performance and investigate between-group comparisons. Functional magnetic resonance imaging should be employed to investigate the effects of finger tapping on brain activities. Second, the participants were classified as having MCI and as healthy participants according to the results of the MoCA-J. Targeting patients with a specific diagnosis can improve the reliability of the results. Finally, owing to the short breaks between the tasks, in future studies, the number of measurements should be increased and thorough comparisons between the tasks should be made.

## 5. Conclusions

This study aimed to examine the extent to which age and MCI affect cerebral blood flow, which was measured using NIR spectroscopy during category, finger-tapping, and dual tasks. Additionally, the effectiveness of cerebral oxyhemoglobin signal measurements in categorizing elderly participants with and without MCI was examined. Our results show that the oxyhemoglobin levels during these tasks were not able to distinguish between elderly participants with and without MCI. However, in participants both with and without MCI, the results suggest that finger-tapping, as well as verbal fluency tasks, can also be effective in activating the prefrontal cortex.

## Figures and Tables

**Table 1:** Participant characteristics.

	Young Control Group (*n* = 30)	Elderly Participants without MCI (*n* = 41)	Elderly Participants with MCI (*n* = 61)	*F* Value
Age (years)	20.9 ± 0.7	74.4 ± 7.1	78.2 ± 6.4	433.6 ***
Sex (M/F)	14:16	9:32	19:42	
MoCA total score (/30)	---	27.5 ± 1.3	21.6 ± 3.1	
Category fluency task performance	7.4 ± 1.3 (7.0–7.9)	6.1 ± 1.8 (5.6–6.5)	4.7 ± 2.1 (4.2–5.2)	21.794 ***
Finger-tapping performance (count)	47.9 ± 9.9 (44.2–51.6)	32.2 ± 11.3 (28.6–35.8)	26.0 ± 11.0 (23.3–29)	40.669 ***
Category fluency task performance (dual task)	7.0 ± 1.2 (6.6–7.5)	5.8 ± 2.1 (5.2–6.5)	4.5 ± 2.0 (3.9–5.0)	19.605 ***
Finger-tapping performance (dual task)	19.7 ± 7.8 (23.3–29.0)	24.5 ± 9.6 (21.4–27.5)	37.4 ± 11.1 (33.2–41.5)	37.339 ***

*** *p* < 0.001; MCI, mild cognitive impairment; MoCA, Montreal Cognitive Assessment; The values in the table represent the mean ± standard deviation (confidence interval).

**Table 2:** Results of the normalized oxyhemoglobin signal area under the curve in the elderly participants with MCI/young control group and without MCI.

	AUC (Elderly Participants with MCI/Young Control Group) (95% CI)	AUC (Elderly Participants with MCI/without MCI) (95% CI)
Right channel during the category fluency task	0.512 (0.375–0.649)	0.496 (0.381–0.611)
Left channel during the category fluency task	0.434 (0.296–0.573)	0.469 (0.350–0.587)
Right channel during the dual task	0.507 (0.378–0.636)	0.570 (0.457–0.684)
Left channel during the dual task	0.499 (0.361–0.638)	0.463 (0.345–0.582)
Right channel during the finger-tapping task	0.607 (0.476–0.737)	0.411 (0.297–0.524)
Left channel during the finger-tapping task	0.641 (0.511–0.771)	0.483 (0.368–0.598)

AUC, area under the curve; CI, confidence interval; MCI, mild cognitive impairment.

## Data Availability

The data sets used and analyzed in the current study are available from the corresponding author upon reasonable request.
